# Cystic mass of the floor of the mouth

**DOI:** 10.4317/jced.54604

**Published:** 2018-03-01

**Authors:** Aina Brunet-Garcia, Elizabeth-Daniela Lucena-Rivero, Laia Brunet-Garcia, Marta Faubel-Serra

**Affiliations:** 1 MD, Department of Otorhinolaryngology, Hospital General Universitari de Castelló. Castelló de la Plana, Universitay of València, València, Spain; 2 MD, Department of Otorhinolaryngology, Hospital Francesc de Borja. Gandia, Spain; 3 MD, Department of Paediatric Cardiology, Hospital Sant Joan de Déu, University of Barcelona, Barcelona, Spain; 4 MD, PhD. Head of Otorhinolaryngology department, Hospital General Universitari de Castelló. Castelló de la Plana, Spain

## Abstract

**Background:**

Epidermoid and dermoid cysts in the oral cavity are relatively uncommon lesions of developmental origin. They often remain asymptomatic for years until they grow enough to interfere with speech, deglutition and less often with breathing which can pose a critical risk to the airway and require immediate surgery.

**Case description:**

A case of an epidermoid cyst of the floor of the mouth affecting a 37-year-old man is presented; this lesion was surgically enucleated with an intraoral approach. Patient did well postoperatively and there was no evidence of recurrence up to 2 years of follow up.

**Clinical implications:**

Floor of the mouth is a challenging site for the diagnosis of a broad variety of lesions which the surgeon should be aware. Depending on the anatomical relation to the muscles of the floor of the mouth dermoid cysts are classified as supramylohyoid or inframylohyoid, and they will both have different clinical and radiological features. This article also includes literature review about the etiopathological, clinical, radiological and histological features, the differential diagnosis and its treatment.

** Key words:**Epidermoid cyst, dermoid cyst, floor of mouth.

## Introduction

Dermoid cysts (DC), epidermoid cysts and teratomas are three histologically closed related cysts englobed in the concept of dermoid cyst. They are most probable located in regions where embryonic elements fuse together, especially in sacral region and ovaries; however, less than 7 percent of DC involve the head and neck region (1-3). An epidermoid cyst should not go unmentioned in the differential diagnosis of a mass of the floor of the mouth.

The aim of this article is to report a case of a 43-year-old man with dermoid cyst located in the floor of the mouth. The typical features of this type of cyst, including etiopathogenesis, radiological and histological findings, the differential diagnosis and its treatment, will also be reviewed.

## Case Report

A 43-year-old man reported to our department with a 2-year history of swelling below tongue and difficulty of speech, mastication, and swallowing. He did not report any pain or dyspnea. Physical examination revealed three centimetres swelling in the floor of the mouth. On palpation, it was non-tender, freely mobile and doughy in consistency. There was mild superiorly and posteriorly displacement of the tongue. Partial drainage was carried out to let the patient eat properly until the lesion could be completely removed.

**Figure 1:**
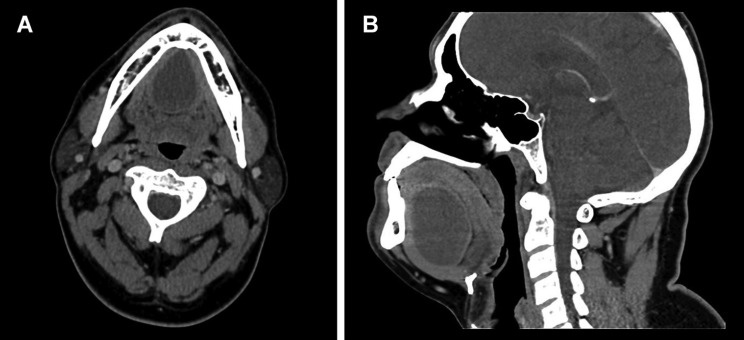
CT scan showing a well-circumscribed lesion in the sublingual space. a) Axial view. b) Sagittal view.

Computed Tomography (CT) showed a 3.9 x 3.1 x 3.4 well-circumscribed lesion that laid within the sublingual space with no extensions through the mylohyoid muscle. There was no evidence of bone involvement (Fig. 1).

**Figure 2:**
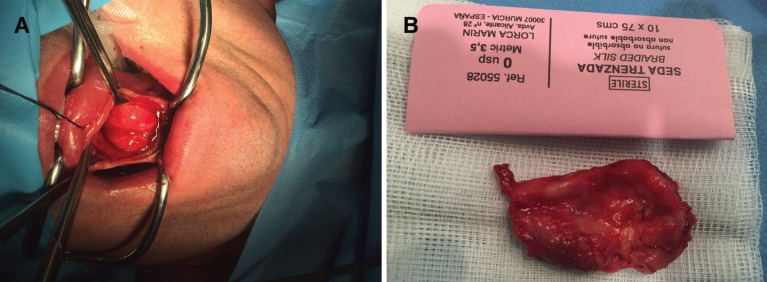
Intraoperative images. A) Exposure of the lesion with a lingual vestibule incision. B) Macroscopic image of the surgical specimen (4 x 3.2 x 3.4 cm).

**Figure 3:**
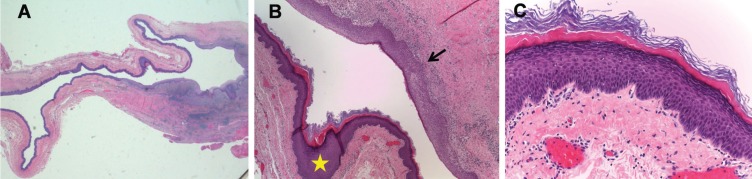
Histopathological examination images. a) H&E Cyst lined by stratified epithelium. b) H&E Chronic inflammatory cells (arrow) and hyperkeratosis (star) (10X). c) H&E Hyperkeratosis with acanthosis (40X).

The lesion was excised under general anesthesia. The incision was performed in lingual vestibule and, to avoid rupture, it was decompressed by aspirating some of the cystic content. Posteriorly, the lesion was enucleated and the surgical wound was closed in layers (Fig. 2). The postoperative healing remained uneventful. The histopathologic exam revealed a dermoid cyst (Fig. 3).

## Discussion

Soft tissue enlargements of the floor of the mouth and submandibular area may be generated by various pathologic processes which can be broadly classified as inflammation, developmental anomalies, cysts and neoplasms (5,6).

Dermoid cyst, epidermoid cyst and teratoma are three histologically closed uncommon lesions englobed in the concept of dermoid cyst. Nonetheless they can be found anywhere in the body they are more probable to be located in regions where embryonic elements fuse together as the ovaries and sacral region.

In a review of 1,495 cases of dermoid cysts, New and Erich found only a 6.94 percent of DC involving the head and neck region and between them, the most common location corresponded to the lateral eyebrow, followed by the floor of the mouth in the 11 percent of the cases (1-3), as in the our patient. DC may occur at any age albeit there is a peak in patients between 15 and 35 years of age with no gender predilection (7).

DC are believed to be originated during the fifth week of embryological development by inclusion of different tissue sources (endoblastic, mesoblastic or ectoblastic) due to a defect during midline union of the first (mandibular) and second (hyoid) branchial arches (3,6,8,9). Traumatic and thyroglossal anomaly theories have also been postulated for pathogenesis.

Meyer described three histological varieties: Epidermoid cyst is lined by simple squamous epithelium without adnexal structures. True dermoid cyst additionally contains skin appendages (sebaceous and sweet glands, hairs and hair follicles in the skin wall). And, the teratoid cyst, also known as complex cyst, may exhibit from simple squamous to ciliated epithelium, containing structures derived from all three germ layers: ectoderm, mesoderm and endoderm (3,4,8).

Depending on the anatomical relation to the muscles of the floor of the mouth, DC are classified as supramylohyoid (intraoral or sublingual), located above mylohyoid and genioglossus muscles and presented as swelling in the floor of the mouth; inframylohyoid, (cervical) between the mylohyoid and geniohyoid, presented as swelling below the chin, also known as “double chin”; and peri- and trans-mylohyoid (both intraoral and cervical) (3,8,10). In our case the patient presented with swelling in the floor of the mouth and a posterosuperior tongue displacement, so that we can classify it as a supramylohyoid cyst.

Clinically, DC present as a slow-growing, painless, midline suprayoid, neck mass (85-90%) (5). In our case the patient presented with speech disorders. They are mostly described to be asymptomatic until they are large enough to cause symptoms as dysphagia and rarely airway infringement owing to its great size. It can acutely grow, due to infection, during pregnancy (because of an increase in sebum production or in plasma levels of estrogens and progesterone that act as growth factors on the cyst) or in association with a sinus tract (2,4).

A broad variety of conditions should be kept in mind for the differential diagnosis. Some of them are thyroglossal or branchial cleft cyst, lipoma, infectious processes of perioral tissues or salivary glands, congenital lesions such as vascular malformation or lymphangioma, Wharton’s duct blockage or malignant soft tissue tumors (6,8).

To rule out these conditions, careful physical examination and other procedures such as fine needle aspiration and diagnostic imaging should be performed. Fine needle aspiration is considered a safe, cost effective and reliable method but not as much as Computed tomography (CT) or Magnetic Resonance Imaging (MRI).

Contrast-enhanced CT is the best diagnostic method except in those patients that have dental amalgam; in which case, MRI is preferred (4).

Furthermore, they are both essential prior to planning surgical approach. CT can distinguish between solid and cystic lesions, giving detailed information about its relation to other structures; DC appear as low-density, well-circumscribed, unilocular mass in the floor of the mouth (8,9).

US shows the presence of posterior acoustic enhancement in cystic lesions as well as its tendency to have a “pseudosolid” appearance.

Treatment consists of surgical removal with complete enucleation by meticulous dissection. Intraoral approach is preferred in cysts superior to mylohyoid muscle as in our case, whereas those occurring below the muscle may require extraoral approach (2,8).

Malignant transformation of sublingual dermoid and epidermoid cysts has been reported along with a 5% of the teratoid variety of oral dermoid cyst cases (3).

## Conclusions

Floor of the mouth is a challenging site for the diagnosis of a broad variety of lesions that extend from benign to malign lesions. Therefore, they can compress the airway or route infections until the mediastinum and hence, contribute to morbidity and mortality. A preoperative evaluation of the cyst’s location in relation to the geniohyoid and mylohyoid muscles, in a sublingual or submental position, is crucial in determining whether to approach the cyst through an intraoral or an extraoral incision. Knowing the diagnosis, histopathological findings, and treatment of these lesions is essential for the management of these patients.
